# Commentary: Association of Umbilical Cord Milking vs. Delayed Umbilical Cord Clamping With Death or Severe Intraventricular Hemorrhage Among Preterm Infants

**DOI:** 10.3389/fped.2020.00178

**Published:** 2020-04-17

**Authors:** Simone Pratesi, Carlo Dani

**Affiliations:** Neonatal Intensive Care Unit, Careggi University Hospital, Florence, Italy

**Keywords:** preterm infant, cord milking, delayed cord clamping, intraventricular hemorrhage, neonatal resuscitation

The quality of care in the first “golden” minutes of life of a newborn, especially if born preterm, can profoundly influence later-life outcomes. A meta-analysis of 12 randomized controlled trials demonstrated that placental transfusion strategies (both delayed cord clamping and cord milking performed at birth) vs. immediate cord clamping significantly reduced intraventricular hemorrhage and mortality in preterm newborn less than 31 weeks gestation (1). Two meta-analyses demonstrated that cord milking or delayed cord clamping compared with immediate cord clamping in premature newborns was associated with a reduced risk of all-grade intraventricular hemorrhage or mortality, respectively (2, 3). Thus, both delayed cord clamping and cord milking at birth seem to be protective compared with immediate cord clamping in preterm newborns. Instead, immediate cord clamping is still a routine care at birth performed worldwide, above all in very preterm newborns.

Recently Katheria and colleagues early terminated a non-inferiority randomized clinical trial of preterm infants receiving placental transfusion with umbilical cord milking vs. delayed umbilical cord clamping, as cord milking was associated with a higher rate of severe intraventricular hemorrhage compared with delayed cord clamping (22 vs. 6%), among infants born at 23 to 27 weeks' gestation (4). Do the results reported by Katheria et al. indicate a clinical harm of milking the cord or, instead, a protective role of delaying (for at least 60 s, with stimulation of the baby) cord clamping at birth? The reported 22% rate of severe intraventricular hemorrhage in milked babies is not surprising as it is compatible with rates reported in Vermont Oxford Network database in newborns less than 27 weeks gestation (17 and 36% at 24–26 w and <24 w gestation, respectively), presumably immediately clamped at birth. Instead, an incidence of severe intraventricular hemorrhage of 3% (and 6% in the 23–27 w gestation subgroup), lower than the 4.5% (9 studies, *n* = 992) observed in the recently published Cochrane review (5), is quite unexpected. This could be due to the “highest rate of adherence to delayed umbilical cord clamping of any multicenter umbilical cord management trial to date” (4): more than 90% of babies had their cord clamped after at least 60 s while dried and given gentle tactile stimulation to promote respiratory effort. It would be important to know how was the intraventricular hemorrhage rate in the group of babies excluded from the study, and immediately clamped at birth, as it could be equal or even worse than in milked babies. However, the reason of the exclusion could select a population with a different (increased or decreased) risk of intraventricular hemorrhage, thus still leaving us with questions concerning what is the correct comparison with regards to severe intraventricular hemorrhage in this population. The best way to answer would probably be to conduct a 3-arm RCT to determine whether the rate of severe intraventricular hemorrhage differ among preterm infants receiving at birth umbilical cord milking vs. delayed umbilical cord clamping vs. immediately cord clamping. Milking the cord in anesthetized preterm lambs determines potentially harmful hemodynamic changes (no increase in pulmonary blood flow and fluctuations in carotid artery flow) similar to those occuring after immediate cord clamping at birth, while no hemodynamic changes occur if the animal breathes before being clamped (6). The more physiological approach of stimulating the start of breathing during the first at least 60 s of life before clamping the cord of very preterm newborns might result neuroprotective, reducing the intraventricular hemorrhage rate compared with cord milking. But, we need randomized controlled trials to demonstrate it.

## Author Contributions

SP and CD conceived of the presented idea. Both authors contributed to the final manuscript.

## Conflict of Interest

The authors declare that the research was conducted in the absence of any commercial or financial relationships that could be construed as a potential conflict of interest.
